# Risk of Fracture After Bilateral Oophorectomy

**DOI:** 10.1002/jbm4.10750

**Published:** 2023-05-01

**Authors:** Trine K Hueg, Martha Hickey, Astrid L Beck, Louise F Wilson, Cecilie S Uldbjerg, Lærke Priskorn, Julie Abildgaard, Youn‐Hee Lim, Elvira V Bräuner

**Affiliations:** ^1^ Department of Growth and Reproduction Copenhagen University Hospital – Rigshospitalet Copenhagen Denmark; ^2^ International Centre for Research and Research Training in Endocrine Disruption of Male Reproduction and Child Health (EDMaRC) Copenhagen University Hospital – Rigshospitalet Copenhagen Denmark; ^3^ Department of Obstetrics and Gynaecology University of Melbourne Melbourne Australia; ^4^ NHMRC Centre for Research Excellence on Women and Non‐communicable Diseases (CREWaND), School of Public Health The University of Queensland Herston Australia; ^5^ Centre for Physical Activity Research Rigshospitalet, University of Copenhagen Copenhagen Denmark; ^6^ Section of Environmental Health, Department of Public Health University of Copenhagen Copenhagen Denmark; ^7^ Seoul National University Medical Research Center Seoul Republic of Korea

**Keywords:** EPIDEMIOLOGY, FRACTURE RISK ASSESSMENT, GENERAL POPULATION STUDIES, HORMONE REPLACEMENT, MENOPAUSE

## Abstract

Fragility fractures, resulting from low‐energy trauma, occur in approximately 1 in 10 Danish women aged 50 years or older. Bilateral oophorectomy (surgical removal of both ovaries) may increase the risk of fragility fractures due to loss of ovarian sex steroids, particularly estrogen. We investigated the association between bilateral oophorectomy and risk of fragility fracture and whether this was conditional on age at time of bilateral oophorectomy, hormone therapy (HT) use, hysterectomy, physical activity level, body mass index (BMI), or smoking. We performed a cohort study of 25,853 female nurses (≥45 years) participating in the Danish Nurse Cohort. Nurses were followed from age 50 years or entry into the cohort, whichever came last, until date of first fragility fracture, death, emigration, or end of follow‐up on December 31, 2018, whichever came first. Cox regression models with age as the underlying time scale were used to estimate the association between time‐varying bilateral oophorectomy (all ages, <51/≥51 years) and incident fragility fracture (any and site‐specific [forearm, hip, spine, and other]). Exposure and outcome were ascertained from nationwide patient registries. During 491,626 person‐years of follow‐up, 6600 nurses (25.5%) with incident fragility fractures were identified, and 1938 (7.5%) nurses had a bilateral oophorectomy. The frequency of fragility fractures was 24.1% in nurses who were <51 years at time of bilateral oophorectomy and 18.1% in nurses who were ≥51 years. No statistically significant associations were observed between bilateral oophorectomy at any age and fragility fractures at any site. Neither HT use, hysterectomy, physical activity level, BMI, nor smoking altered the results. © 2023 The Authors. *JBMR Plus* published by Wiley Periodicals LLC on behalf of American Society for Bone and Mineral Research.

## Introduction

Fragility fractures result from low‐energy trauma fractures such as falling from standing height or less.^(^ ^1^ ^)^ The incidence in Denmark is almost 10% in women aged 50 years or older.^(^ ^2^ ^)^ Fragility fractures have a negative impact on future health‐related quality of life, including impaired mobility and self‐care^(^ ^3^ ^)^ and constitute an increased risk of future fractures^(^ ^4^ ^)^ and mortality.^(^ ^5^ ^)^ Given the aging population in high‐income countries, fragility fractures are considered a significant public health issue.^(^ ^6^, ^7^ ^)^

Bilateral oophorectomy is a surgical procedure where both ovaries are removed. In some cases, bilateral oophorectomy is performed as a risk‐reducing procedure in women with high inherited risk of ovarian cancer.^(^ ^8^ ^)^ Premenopausal bilateral oophorectomy is associated with a significant reduction in circulating sex steroids, including estrogen, progesterone, and testosterone.^(^ ^9^ ^)^ Sex steroids, particularly estradiol, contribute to bone health, and a reduction in circulating estrogen is associated with greater bone resorption than bone formation, which is a major risk factor of fragility fractures.^(^ ^10^ ^)^ Several studies have previously demonstrated a clear association between bilateral oophorectomy and loss of bone mass,^(^ ^11^, ^12^, ^13^ ^)^ but only a few studies have specifically investigated the relationship between bilateral oophorectomy and fragility fractures, with inconclusive findings.^(^ ^14^, ^15^, ^16^, ^17^ ^)^ Around half of these studies suggested an increased risk,^(^ ^14^, ^15^, ^17^ ^)^ whereas others showed no association or reduced risk.^(^ ^16^, ^18^, ^19^ ^)^ Differences in the reported results from previous studies imply that any associations may be conditional on age or menopausal status at the time of bilateral oophorectomy as well as the site‐specific fracture type. Importantly, the previous studies reporting on the association between bilateral oophorectomy and fragility fracture are generally limited by small study populations (*n* < 500) and short follow‐up periods.

Previous studies investigating the effect of hormone therapy (HT) on fracture risk, including two observational studies and two randomized controlled studies, have demonstrated a protective effect of HT on fracture of the spine,^(^ ^20^, ^21^ ^)^ hip,^(^ ^20^, ^21^, ^22^ ^)^ and forearm^(^ ^20^, ^23^ ^)^ in postmenopausal women from the general population. However, whether use of HT modifies the association between bilateral oophorectomy and fragility fracture is uncertain. To date, no studies have investigated the modifying effect of HT on the association between bilateral oophorectomy and fragility fracture. Hysterectomy is often performed at time of bilateral oophorectomy, and hysterectomy with ovarian conservation reduces age at menopause.^(^ ^24^ ^)^ However, the evidence diverges regarding the effect of hysterectomy on fracture risk.^(^ ^25^, ^26^ ^)^ Further, modifiable factors such as physical activity level,^(^ ^27^, ^28^ ^)^ body mass index (BMI),^(^ ^29^ ^)^ and smoking^(^ ^30^, ^31^ ^)^ may affect the risk of fragility fracture, but no studies have investigated the modifying effects of physical activity level, BMI, or smoking on the association between bilateral oophorectomy and fragility fracture.

In this study, we aimed to investigate the risk of fragility fracture after bilateral oophorectomy and whether this association is conditional on age at time of bilateral oophorectomy, HT use, hysterectomy, physical activity level, BMI, or smoking.

## Methods

### Study design and data source

**Fig. 1:**
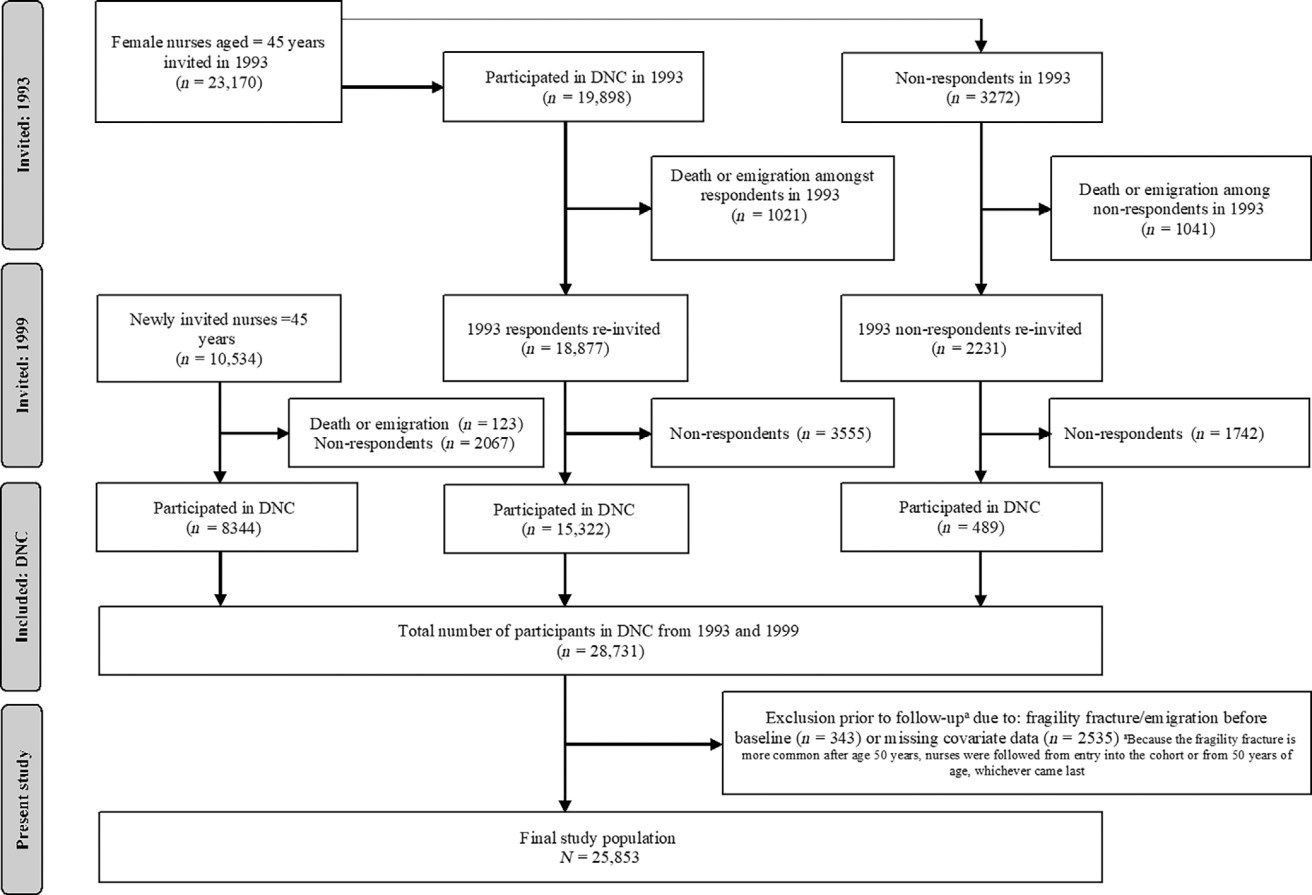
Danish Nurse Cohort (DNC; invitations, exclusions, participation) and present study (exclusion, participation).

We applied a prospective cohort study design using the Danish Nurse Cohort, established in 1993^(^ ^32^ ^)^ and comprising female nurses recruited from the Danish Nurse Organization. In 1993, 23,170 female members of the Danish Nurse Organization (≥45 years) were invited to participate, whereof 19,898 (86%) accepted. In 1999, the Danish Nurse Cohort was reinvestigated, and an additional 8833 (69%) female Danish Nurse Organization members were included (including newly invited nurses who turned 45 years in the interim since 1993 [*n* = 8344] and non‐respondents from 1993 [*n* = 489]). A total of 28,731 female nurses aged ≥45 years were recruited to the cohort (Fig. 1).^(^ ^32^, ^33^, ^34^, ^35^, ^36^, ^37^ ^)^

At study entry, the included nurses completed a self‐administered questionnaire with information on lifestyle, self‐reported height and weight, and previous and current use of HT. The self‐reported HT use has previously been validated, and researchers found high to moderate sensitivity (78.4%) and specificity (98.4%) of self‐reported use of HT compared with registration in the Danish Prescription Registry.^(^ ^38^ ^)^ If nurses participated in both 1993 and 1999, the 1993 questionnaire record was used as baseline. Nurses who completed the baseline questionnaire were linked to Danish national registries including the Danish Civil Registration System and the National Patient Registry using a unique identification number assigned to all Danish citizens.

### Study population

Of the 28,731 nurses included in the Danish Nurse Cohort, 2878 were excluded because of incident fragility fracture or emigration before baseline (*n* = 343) or missing covariate data (*n* = 2535). The final study population included 25,853 nurses for complete case analysis (Fig. 1).

### Bilateral oophorectomy exposure

Ascertainment of bilateral oophorectomy has previously been described in detail.^(^ ^33^ ^)^ In brief, bilateral oophorectomy procedures were identified in the National Patient Registry using International Classification of Disease (ICD) 8th (before 1993) and 10th (1993 and onward) revision procedure codes, where the exact date of the procedure is recorded.^(^ ^33^ ^)^ Time‐variant bilateral oophorectomy was defined based on the date of the procedure. Unexposed nurses (both ovaries preserved) contributed person‐years until the date of their first oophorectomy. Nurses with a bilateral oophorectomy before baseline entered the model as exposed. Nurses who had two consecutive unilateral oophorectomies performed (*n* = 101) changed exposure status from unilateral to bilateral oophorectomy on the date of their second unilateral oophorectomy. Nurses with preserved ovaries were the reference group. Time‐variant unilateral oophorectomy was considered in the model as a covariate (cf. covariate ascertainment described below) based on the time of the procedure.

The associations between bilateral oophorectomy and fragility fracture were examined for the total study population (all ages) and according to age at surgery for bilateral oophorectomy using a dichotomized variable (<51/≥51 years) as proxy for pre‐ and postmenopausal status, respectively, based on the average of the median age of menopause reported in two European studies (50.1 and 52.8 years).^(^ ^39^, ^40^ ^)^

### Fragility fracture ascertainment

Incident fragility fracture was ascertained from the National Patient Registry using ICD 8 and 10 procedure codes. In Denmark, fractures reported in the registry are always confirmed by an X‐ray or computer tomography scan. The registry does not provide cause of fracture, which complicates the distinction between fragility fracture and non‐fragility fractures. To limit misclassification, we proceeded with the following steps as described previously in three Danish studies relating to fragility fracture.^(^ ^41^, ^42^, ^43^ ^)^ First, fractures recognized to be the result of high‐energy trauma (hand/fingers, foot/toes, head/skull, multiple) were not considered. Second, given that fragility fractures are most prevalent after age 50 years,^(^ ^7^ ^)^ we followed nurses from age 50 years or entry into the cohort, whichever came last. Fracture risk was investigated as any and site‐specific (spine, hip, forearm, other) incident fragility fractures of interest separately. ICD codes of included fragility fractures are available in Supplemental Table S1.

### Covariate ascertainment

Potential confounders were identified a priori using a causal diagram based on a review of the literature of risk factors of bilateral oophorectomy and fragility fracture. Potential confounding variables were obtained from the self‐reported baseline questionnaire, including BMI (<25, ≥25 kg/m^2^), smoking status (never, previous, current), alcohol consumption (none, low ≤7, moderate 8–14, high >14 units/week), physical activity level (low [sedentary], moderate [light exercise ≥4 h/week], high [frequent elite sports or heavy lifting]), and use of HT reported at baseline (never, ever). Unilateral oophorectomies and hysterectomy were identified using ICD procedure codes recorded in the National Patient Registry and included as time‐varying covariates.^(^ ^33^ ^)^

### Statistical analyses

Descriptive statistics (median with 5th–95th percentile or frequencies) were calculated for all included variables stratified according to oophorectomy status (none, unilateral, or bilateral).

In our main analyses, we applied time‐varying Cox regression models with age as the underlying timeline to investigate the association between bilateral oophorectomy (all ages and stratified by age at time of bilateral oophorectomy) and risk of incident fragility fracture (any and site‐specific [hip, spine, forearm, other]) compared with referent nurses with retained ovaries. Nurses were followed from 50 years of age or study entry until date of incident fragility fracture (any and site‐specific fracture in separate models), death, emigration, or end of follow‐up (December 31, 2018), whichever came first. Results are presented as hazard ratios (HR) with 95% confidence interval (CI) for any and site‐specific fragility fracture. We constructed two models with a priori determined confounders: Model 1 was adjusted for calendar year and time‐variant unilateral oophorectomy. Model 2 was further adjusted for BMI, smoking status, alcohol intake, physical activity level, HT, and time‐variant hysterectomy. Bilateral oophorectomy entered the model as a time‐varying variable based on date of procedure. Unilateral oophorectomy was analyzed as a separate group (data are not shown).

The potential modifying effects of HT, hysterectomy, physical activity level, BMI, or smoking on the association between bilateral oophorectomy and any fragility fractures were estimated by comparing risk of any fragility fractures in nurses with and without bilateral oophorectomy within levels of each potential effect modifier using the likelihood ratio test for interaction in model 2 but with no adjustment for the interacting variable.

Cox regression models were estimated using the PHREG procedure in SAS version 9.4, statistical software package (SAS Institute, Cary, NC, USA). All statistical tests were two‐sided, and *p* values <0.05 were considered statistically significant.

The study follows the Enhancing the Quality and Transparency of Health Research (EQUATOR) reporting guidelines for observational studies (STROBE).^(^ ^44^ ^)^

### Ethical considerations

The authors assert that all procedures contributing to this work comply with the ethical standards of the relevant national and institutional committees on human experimentation and with the Helsinki Declaration. The present study was approved by the Danish Data Protection Agency (j.nr. VD‐2018‐451, suite nr. 06707), and the nurses in the Danish Nurse Cohort provided informed written consent. Furthermore, Danish Nurse Cohort inclusion was approved by local Danish Ethical Committee (J.nr. BFH‐2019‐001, suite nr. 06102).

## Results

### Basic characteristics

During 491,626 person‐years of follow‐up, a total of 6660 (25.8%) nurses were registered with an incident fragility fracture. Characteristics of the study population stratified by oophorectomy status are presented in Table 1. Of 25,853 included nurses, 1938 (7.5%) had a bilateral oophorectomy. In general, the proportion of obesity (BMI ≥30), non‐smoking, ever use of HT, and hysterectomy was higher in nurses with bilateral oophorectomy compared with nurses with preserved ovaries (*n* = 22,960) or nurses with unilateral oophorectomy (*n* = 955) (Table 1). The frequency of any fragility fracture was 26.5% in nurses with preserved ovaries and 19.4% in nurses with a bilateral oophorectomy (Table 1). The most common fragility fracture was forearm, and the frequency of any fragility fracture was 24.1% in nurses <51 years at time of bilateral oophorectomy (proxy for premenopausal) and 18.1% in nurses ≥51 years at time of bilateral oophorectomy (proxy for postmenopausal) (Table 1).

**Table 1:** Person‐Related Characteristics for the 25,853 Female Nurses (Danish Nurse Cohort), Stratified by Oophorectomy Status

Baseline characteristics	Oophorectomy		
	None (*n* = 22,960)	Unilateral (*n* = 955)	Bilateral (*n* = 1938)
Age (years), median (5th–95th percentile)	50.4 (44.9–70.3)	49.6 (44.9–65.0)	50.6 (45.0–68.7)
Body mass index (BMI) (kg/m^2^), *n* (%)			
<18.5	589 (2.5)	22 (2.3)	28 (1.4)
18.5–24.9	15,861 (69.1)	643 (67.3)	1307 (67.4)
25–29.9	5251 (22.9)	236 (24.7)	447 (23.1)
≥30.0	1259 (5.5)	54 (5.7)	156 (8.1)
Smoking status, *n* (%)			
Never	7824 (34.1)	316 (33.1)	711 (36.7)
Previous	7020 (30.6)	288 (30.2)	609 (31.4)
Current	8116 (35.3)	351 (36.7)	618 (31.9)
Alcohol consumption (units/week), *n* (%)^a^			
None	3592 (15.6)	140 (14.7)	322 (16.6)
Low drinker (≤7)	8507 (37.1)	348 (36.4)	706 (36.4)
Moderate drinker (8–14)	5618 (24.5)	229 (24.0)	469 (24.2)
Heavy drinker (>14)	5243 (22.8)	238 (24.9)	441 (22.8)
Physical activity level, *n* (%)			
Low (sedentary)	1559 (6.8)	55 (5.7)	120 (6.2)
Moderate (light exercise ≥4 h/week)	15,220 (66.3)	633 (66.3)	1343 (69.3)
High (frequent elite sports or heavy lifting)	6181 (26.9)	267 (28.0)	475 (24.5)
Hormone therapy, *n* (%)			
Never	17,080 (74.4)	605 (63.4)	1031 (53.2)
Ever	5880 (25.6)	350 (36.6)	907 (46.8)
**Time‐varying variables, diagnosed during follow‐up**			
Any fragility fractures, *n* (%)^b^	6076 (26.5)	208 (21.8)	376 (19.4)
Spine	352 (1.5)	15 (1.6)	21 (1.1)
Forearm	3253 (14.2)	114 (11.9)	179 (9.2)
Hip	1397 (6.1)	32 (3.4)	93 (4.8)
Other fragility fractures	1074 (4.7)	47 (5.0)	83 (4.4)
Osteoporosis, *n* (%)	1642 (7.2)	85 (8.9)	124 (6.4)
Hysterectomy, *n* (%)	2185 (9.5)	408 (42.7)	1590 (82.0)
Bilateral oophorectomy, *n* (%)			
<51 years^c^	NA	NA	419 (21.6)
≥51 years^d^	NA	NA	1,519 (78.4)
Unilateral oophorectomy, *n* (%)			
<51 years	NA	636 (66.6)	NA
≥51 years	NA	319 (33.4)	NA

^a^ Including beer (regular and strong), wine (red and white), and liquor.

^b^ Fractures diagnosed ≥50 years were considered.

^c^ Total fragility fracture in premenopausal women, *n* = 101 (24.1%).

^d^ Total fragility fracture in postmenopausal women, *n* = 275 (18.1%).

### Associations between bilateral oophorectomy and fragility fractures

No statistically significant associations were observed for nurses <51 years of age at the time of bilateral oophorectomy and any fragility fracture (aHR = 1.12; 95% CI, 0.91–1.39), forearm fracture (aHR = 1.15; 95% CI, 0.86–1.54), hip fracture (aHR = 1.15; 95% CI, 0.69–1.92), spine fracture (aHR = 0.98; 95% CI, 0.42–2.32), and other fracture (aHR = 1.21; 95% CI, 0.89–1.64) compared with nurses with retained ovaries. Similarly, no statistically significant associations were observed between bilateral oophorectomy (all ages and ≥51 years of age) and fragility fractures with magnitudes of the estimates close to unity. Estimates in crude and adjusted models were similar (Table 2).

**Table 2:** Hazard Ratios (HR) and 95% Confidence Interval (CI) of Incident Fragility Fracture (Diagnosed ≥50 Years) in Nurses From the Danish Nurse Cohort (*n* = 25,853) With Bilateral Oophorectomy (All Ages and Stratified by Age [<51 Years and ≥51 Years]) at Time of Oophorectomy as Proxy of Menopausal Status) Compared With Referent Women With No Oophorectomy

Bilateral oophorectomy	Fragility bone fracture type	*N*_fracture cases_	HR (95% CI)	
			Model 1^a^	Model 2^b^
Premenopausal bilateral oophorectomy (<51 years)	Any fragility fracture	101	1.07 (0.88–1.30)	1.12 (0.91–1.39)
	Spine	6	1.10 (0.49–2.47)	0.98 (0.42–2.32)
	Forearm	53	1.01 (0.77–1.32)	1.15 (0.86–1.54)
	Hip	17	1.10 (0.68–1.78)	1.15 (0.69–1.92)
	Other	25	1.28 (0.96–1.70)	1.21 (0.89–1.64)
Postmenopausal bilateral oophorectomy (≥51 years)	Any fragility fracture	275	0.92 (0.81–1.04)	0.95 (0.83–1.09)
	Spine	15	0.77 (0.46–1.29)	0.76 (0.43–1.37)
	Forearm	126	0.85 (0.71–1.01)	0.93 (0.76–1.13)
	Hip	76	0.96 (0.77–1.20)	0.99 (0.75–1.31)
	Other	58	0.97 (0.81–1.16)	0.93 (0.75–1.14)
All ages	Any fragility fracture	376	0.95 (0.86–1.06)	0.98 (0.87–1.11)
	Spine	21	0.85 (0.55–1.32)	0.80 (0.47–1.34)
	Forearm	179	0.89 (0.76–1.03)	0.98 (0.83–1.17)
	Hip	93	0.98 (0.80–1.20)	1.01 (0.78–1.32)
	Other	83	1.03 (0.88–1.21)	0.98 (0.82–1.18)

^a^ Model 1 adjusted for current age and calendar year as an underlying timeline and time‐varying unilateral oophorectomy.

^b^ As for model 1, with further adjustment for body mass index (<18.5, 18.5–24.9, 25–29.9, ≥30 kg/m^2^), smoking status (current, previous, never), alcohol consumption (none, low, moderate, high), physical activity level (low, moderate, high), hormone therapy (never, ever), and hysterectomy (time‐varying).

### Effect modifications

No statistically significant modifying effects of HT (ever, never) (*p*_interaction_ = 0.67), hysterectomy (yes, no) (*p*_interaction_ = 0.15), physical activity level (moderate/high, low) (*p*_interaction_ = 0.67), BMI (<25/≥25 kg/m^2^) (*p*_interaction_ = 0.13), and smoking (ever, never) (*p*_interaction_ = 0.61) on the association between bilateral oophorectomy and any fragility fracture were detected (Table 3).

**Table 3:** Effect Modification of the Association Between Bilateral Oophorectomy (All Ages) and Any Fragility Fracture Risk (Diagnosed ≥50 Years) by Hormone Therapy (HT), Hysterectomy, Body Mass Index (BMI), and Physical Activity Level (PAL) (Women With Both Ovaries Preserved Served as Reference Group)

Effect modifier	Bilateral oophorectomy	HR (95% confidence interval)^a^	*p* Value^b^
HT (never)	No	1 (reference)	0.67
	Yes	1.02 (0.86–1.21)	
HT (ever)	No	1 (reference)	
	Yes	0.97 (0.81–1.17)	
Hysterectomy (no)	No	1 (reference)	0.15
	Yes	1.15 (0.90–1.48)	
Hysterectomy (yes)	No	1 (reference)	
	Yes	0.97 (0.84–1.11)	
PAL (low)	No	1 (reference)	0.67
	Yes	1.07 (0.65–1.76)	
PAL (moderate or high)	No	1 (reference)	
	Yes	0.96 (0.85–1.09)	
BMI (<25 kg/m^2^)	No	1 (reference)	0.13
	Yes	0.89 (0.77–1.02)	
BMI (≥25 kg/m^2^)	No	1 (reference)	
	Yes	1.22 (0.97–1.54)	
Smoking (no)	No	1 (reference)	0.61
	Yes	1.14 (0.93–1.40)	
Smoking (yes)	No	1 (reference)	
	Yes	1.20 (1.03–1.39)	

^a^ Bilateral oophorectomy status entered the model as a time‐varying variable. Models adjusted for age as an underlying timeline, calendar period, BMI (<18.5, 18.5–24.9, 25–29.9, ≥30 kg/m^2^), smoking status (current, previous, never), alcohol consumption (none, low, moderate, high), PAL (low, moderate, high), HT (ever, never), hysterectomy (time‐varying), and unilateral oophorectomy (time‐varying), but with no adjustment for the interaction variable.

^b^ Test of the null hypothesis that the hazard ratios are identical, using a likelihood ratio test.

## Discussion

In this large nationwide prospective registry‐based cohort study of 25,583 nurses, we observed no statistically significant associations between bilateral oophorectomy at any age and fragility fractures at any site. We found no evidence that HT use, hysterectomy, physical activity level, BMI, or smoking altered the association between bilateral oophorectomy and any fragility fracture.

Although there was no statistically significant associations between bilateral oophorectomy at <51 years of age and fragility fracture, the direction of the estimates pointed toward increased risks, which supports the fact that loss of ovarian sex steroids before natural menopause negatively affects bone health.^(^ ^45^, ^46^ ^)^ Similarly, four previous studies report estimates pointing in the direction of either increased or reduced risk of fragility fracture after bilateral oophorectomy, although the associations are statistically insignificant.^(^ ^14^, ^17^, ^18^, ^19^ ^)^ Two small observational studies (*n* < 500) reported a statistically insignificant increased rate of any fragility fracture in women younger than 45 years^(^ ^17^ ^)^ and forearm fracture^(^ ^14^ ^)^ in women aged 45–49 years at time of bilateral oophorectomy compared with expected rates in the general population of women within the same age group. Another observational study of almost 30,000 women in the United States reported a statistically insignificant increased risk of hip fracture after bilateral oophorectomy with hysterectomy at age 45–54 years, which is in line with our finding, but a reduced risk of hip fracture in women aged <45 years with hysterectomy with bilateral oophorectomy compared with women with hysterectomy alone.^(^ ^18^ ^)^ A single study of almost 25,500 women also found a statistically insignificant reduced risk of hip fracture after bilateral oophorectomy with hysterectomy at ≤40 years of age but no effect in those aged 40–49 years compared with women with hysterectomy alone.^(^ ^19^ ^)^ However, because these two large studies included hysterectomy within the strata of both exposed and reference population, we cannot directly compare these previous results^(^ ^18^, ^19^ ^)^ with our findings.

In our study, most nurses were ≥51 years of age at the time of bilateral oophorectomy. The observed results of no association in this group is consistent with a previous study reporting no association of hip fracture in women after postmenopausal bilateral oophorectomy compared with women with preserved ovaries.^(^ ^16^ ^)^ Similarly, a small study reported reduced rates of forearm fracture in women ≥50 years of age at time of bilateral oophorectomy compared with expected rates in the general population of women within the same age group.^(^ ^14^ ^)^ In contrast, a small study of 340 women in the United States found higher than expected rates of hip, forearm, and spine fracture after postmenopausal bilateral oophorectomy.^(^ ^15^ ^)^

In the present study, no statistically significant associations were observed for bilateral oophorectomy (all ages, <51/≥51 years) and spine fractures. Spine fractures were the least prevalent and may be asymptomatic;^(^ ^47^, ^48^ ^)^ hence, complete ascertainment of spine fractures may be less than other fragility fractures, potentially leading to bias in an uncertain direction. Only three previous studies have reported on the association between bilateral oophorectomy and spine fractures. One previous study, in line with ours, found no effect of bilateral oophorectomy at age ≥65 years on spine fractures compared with women without oophorectomy.^(^ ^16^ ^)^ However, two other studies (*n* < 500) detected a statistically significant increased risk of spine fracture after postmenopausal bilateral oophorectomy^(^ ^15^ ^)^ and in women aged 45–49 years at time of bilateral oophorectomy compared with expected rates in the general population of women within the same age group.^(^ ^14^ ^)^

Denmark has a very high rate of major fractures. A systematic review by Kanis and colleagues reported annual age‐standardized incidence rates of hip fracture among women in Denmark of 574 per 100,000 women, followed by Norway at 563 per 100,000 and Sweden at 539 per 100,000.^(^ ^49^ ^)^ The reasons for this high prevalence are not fully known. Initially, these numbers were thought to be explained by genetic factors^(^ ^50^ ^)^ and vitamin D insufficiency,^(^ ^51^ ^)^ but more recent randomized controlled trials found that vitamin D supplements did not improve bone mass density in females aged ≥70 years^(^ ^52^ ^)^ nor reduce fracture risk in women aged 55 years.^(^ ^53^ ^)^ Regardless of the underlying cause of this high prevalence, these unknown risk factors for fragility fracture may mask any potential effect of bilateral oophorectomy on the risk of fragility fracture.

### Strengths and limitations

This prospective longitudinal study utilized a large national cohort. The cohort was well characterized at baseline, had a long follow‐up period, and objectively ascertained exposures and outcome through unique linkage to Danish national registries. All Danish female nurses registered in the Danish Nurse Organization were invited to participate in this cohort, reducing potential selection bias. Data quality and validity are expected to be high, as reporting to the registries is compulsory in Denmark. The Danish Nurse Cohort is also particularly homogenous regarding socioeconomic factors that confound other studies. Also, the Danish population (and nurses included in the Danish Nurse Cohort) is homogenous with 98% being of Caucasian descent; thus, we do not expect ethnicity to affect reported estimates. However, both racial and socioeconomic homogeneity can hamper the ability to generalize findings to populations of other racial and socioeconomic groups.

We limited our analyses to fractures occurring >50 years of age (based on the knowledge that fragility fractures occur more often at >50 years) and we did not consider fractures caused by high‐energy trauma and multiple fractures. However, we cannot with certainty know that this definition covered all fragility fractures, potentially leading to some ascertainment bias.

Oophorectomies performed before the initiation of the National Patient Registry in 1977 would not be registered, causing some potential exposure misclassification. However, we do not expect this to be a major issue as the average age at baseline was approximately 50 years and bilateral oophorectomy <50 years of age is uncommon in Denmark. We stratified oophorectomy by age at surgery (<51/≥51 years of age) as a proxy for pre‐ and postmenopausal status and our age cut‐offs may have grouped women at varied reproductive stages together, possibly causing misclassification within the exposure group.^(^ ^39^, ^40^ ^)^ Potential confounding information including HT use was collected from the self‐reported baseline questionnaire and might have changed during follow‐up, which we were unable to account for. Although we had access to the Danish Prescription Registry, we were unable to utilize time‐varying HT data because this registry was not initiated until 1995 and use of time‐varying HT would further reduce our statistical power. Despite the large population within the Danish Nurses Cohort, our analyses were limited by low statistical power because of the small number of strata‐specific fragility fracture events. Therefore, we were unable to apply 10‐year intervals for age at bilateral oophorectomy or perform the effect modification analyses on subgroups of bilateral oophorectomy, which would have given us a clearer indication of how age modified the association between bilateral oophorectomy and fracture risk. Also, adjusted confounders were primarily dichotomized, leading to loss of precision. Altogether, this affected statistical precision, generally resulting in wide confidence intervals crossing unity, which limited our conclusions.

Finally, there may be a risk of ascertainment bias in women with bilateral oophorectomy, as they may be in more regular contact with the health care system than other women, potentially leading to increased detection of deteriorated bone health and more health care, including treatment for osteoporosis. Together, these would mask or change the true effect of oophorectomy on risk of fragility fracture.

In this large prospective register‐based cohort study, no statistically significant associations were observed between bilateral oophorectomy at any age and fragility fracture at any site compared with referent nurses with retained ovaries. Although the point estimates suggested increased risk in nurses aged <51 years at time of bilateral oophorectomy, limited statistical power hampers statistical precision, with confidence intervals crossing unity. The association between bilateral oophorectomy and any fragility fracture was not modified by use of HT, hysterectomy, BMI, or physical activity level.

## Author Contributions

**Trine K. Hueg:** Conceptualization; data curation; formal analysis; methodology; writing – original draft; writing – review and editing. **Martha Hickey:** Conceptualization; methodology; supervision; writing – original draft; writing – review and editing. **Astrid Beck:** Methodology; writing – original draft; writing – review and editing. **Louise F. Wilson:** Conceptualization; methodology; supervision; writing – original draft; writing – review and editing. **Cecilie S. Uldbjerg:** Methodology; writing – original draft; writing – review and editing. **Lærke Priskorn:** Conceptualization; methodology; supervision; writing – original draft; writing – review and editing. **Julie Abildgaard:** Methodology; supervision; writing – original draft; writing – review and editing. **Youn‐Hee Lim:** Conceptualization; data curation; formal analysis; methodology; supervision; writing – original draft; writing – review and editing. **Elvira V. Bräuner:** Conceptualization; data curation; formal analysis; investigation; methodology; project administration; supervision; writing – original draft; writing – review and editing.

## Disclosures

This research was funded by The Health Foundation of Denmark (Helsefonden, grant no. 19‐B‐0077), which covered salaries for TKH and EVB. MH was funded by the Australian National Health and Medical Research Council (NHMRC). LW was funded by an NHMRC Centres for Research Excellence grant (APP1153420). The funders had no role in the design and conduct of the study; collection, management, analysis, and interpretation of data; preparation, review, or approval of the manuscript; or the decision to submit the manuscript for publication. All other involved authors declare no conflicts of interest.

### Peer Review

The peer review history for this article is available at https://www.webofscience.com/api/gateway/wos/peer-review/10.1002/jbm4.10750.

## Supporting information


**Table S1.** ICD Diagnosis Codes for Included Fragility Fractures
