# Supplementary material for: Risk of Fracture After Bilateral Oophorectomy
Source: JBMR Plus. 2023 May 1;7(7):e10750. doi: 10.1002/jbm4.10750 (PMC10339092; doi:10.1002/jbm4.10750)
Supplement: Supplementary file 1 — Table S1. ICD Diagnosis Codes for Included Fragility Fractures [file JBM4-7-e10750-s001.docx]

| **Table S1.** ICD diagnosis codes for included fragility fractures | | | |
| --- | --- | --- | --- |
| **Fracture group** | **Fracture type** | **ICD 10 codes** | **ICD 8 codes** |
| Spine | Thoracic spine | S22.0* | 80510, 80511, 80519, 80599 |
|  | Lumbar spine | S32.0*, S32.8A |  |
|  | Spine UNS | T08, T08.9 |  |
| Hip |  | S72.0, S72.1*, S72.2 | 82000, 82001, 82002, 82003, 82009 |
| Forarm |  | S52.0*, S52.1*, S52.2, S52.3, S52.5*, S52.8*, S52.9 | 81300, 81301, 81309, 81310, 81311, 81319, 81320, 81328, 81329 |
| Other fragility fractures | Clavicle | S42.0* | 81099 |
|  | Scapula | S42.1 | 81199 |
|  | Humerus | S42.2*, S42.3*, S42.4*, S42.8 | 81200, 81201, 81208, 81209, 81219, 81220, 81221, 81222, 81229 |
|  | Femur | S72.3, S72.4*, S72.8*, S72.9 | 82109, 82110, 82111, 82118, 82119, 82199 |
|  | Pelvis | S32.1, S32.2, S32.3, S32.4, S32.5, S32.8B, S32.8C | 80529, 80800, 80808, 80809 |
|  | Tibia and fibula | S82.0, S82.1*, S82.2, S82.3, S82.3A, S82.4, S82.9 | 82299, 82300, 82301, 82302, 82303, 82304, 82305, 82309, 82310, 82311, 82319, 82329 |
|  | Ribs and sternum | S22.2, S22.3 | 80709, 80719 |
